# Examination of the Therapeutic Potential of Mouse Oral Mucosa Stem Cells in a Wound-Healing Diabetic Mice Model

**DOI:** 10.3390/ijerph17134854

**Published:** 2020-07-06

**Authors:** Shiri Kuperman, Ram Efraty, Ina Arie, Arkadi Rahmanov, Marina Rahmanov Gavrielov, Matityahau Noff, Ron Fishel, Sandu Pitaru

**Affiliations:** 1 School of Dental Medicine, Faculty of Medicine, Tel Aviv University, Tel Aviv 6997801, Israel; ina.arie@gmail.com (I.A.); phenomenar18@gmail.com (A.R.); dr.marindent@gmail.com (M.R.G.); sandu@pitaru.net (S.P.); 2 Shamir Medical Center, Tel Aviv University, Tel Aviv 6997801, Israel; ramief@gmail.com (R.E.); matinoff49@gmail.com (M.N.); 3 Kaplan Medical Center, Hebrew University of Jerusalem, Jerusalem 91905, Israel; dr.fishelron@gmail.com

**Keywords:** oral stem cells, diabetic wound healing model, wound healing, diabetic mice model

## Abstract

Diabetic wounds’ delayed healing response is still considered a major therapeutic challenge. Stem cells and derived cellular products have been an active field of research for novel therapies referred to as regenerative medicine. It has recently been shown that human oral mucosa stem cells (hOMSCs) are a readily accessible source for obtaining large quantities of stem cells. This study evaluates the potential of mouse oral mucosa stem cells (mOMSCs) to enhance wound healing in a diabetic (*db/db*) mouse model by morphological and histological analysis. We show that mOMSCs-treated wounds displayed a significantly faster wound-healing response (*p* ≤ 0.0001), featuring faster re-epithelialization and a larger area of granulation tissue (*p* ≤ 0.05). Taken together, these results suggest that oral mucosa stem cells might have therapeutic potential in diabetic wound healing.

## 1. Introduction

Stem cells and derived products have been for some time the focus for novel therapies for regenerative medicine. The different types of stem cells, such as induced pluripotent stem cells (iPSCs), embryonic stem cells (ESCs), and adult stem cells, vary in origin, genetic, epigenetic, and biochemical characteristics, thus varying in potential therapeutic indications.

In recent years, oral cavity-derived stem cells have gained growing attention. These cells are classified into two categories: dental stem cells, which are found in the dental pulp (DPSCs), apical papilla (stem cells from apical papilla—SCAP) and human exfoliated deciduous teeth (SHED), and non-dental oral stem cells originating either in the gingiva (gingiva mucosal stem cells—GMSCs), periodontal ligament (periodontal ligament stem cells—PDLSCs), dental follicle (dental follicle stem cells—DFSCs), and the human periapical cyst (hPCy-MSCs) [1,2,3]. 

A major clinical advantage of oral stem cells use in medicine is based on the technical ease of cell harvesting as well as facilitated healing of donor sites, compared to other procedures used for harvesting tissues for stem cell generation, i.e., bone marrow SC, mesenchymal adipose tissue SC, etc. 

Recently, our group isolated and characterized a novel oral stem cell source derived from the lamina propria of the oral mucosa, termed human oral mucosa stem cells (hOMSCs). These cells are of primitive neural crest origin and have been shown to differentiate into all three germ layers in vitro. These cells are easily accessible and produce a viable stable culture relatively fast (a small biopsy is sufficient to produce trillions of stem cells) [4,5]. Particularly noteworthy is that in contrast to most oral stem cells, hOMSCs are available throughout the donor’s life, exhibiting similar characteristics when isolated from young or aged donors, and are minimally affected by advanced passages [4]. Taken together, these advantages point to the high potential of these stem cells as an excellent source for regenerative medicine.

Diabetes is a metabolic disease characterized by high blood glucose levels caused by an inability to produce and/or to respond to insulin signaling [6]. Diabetic foot ulcers (DFUs) are one of the leading causes of morbidity and mortality in diabetic patients [7,8]. Decrease in vascular circulation, reduction in blood flow, and decrease in growth factor production are some of the deficits underlying diabetes delayed wound-healing response [9,10]. As currently available therapies for treating diabetic wounds harbor significant drawbacks, researchers have proposed stem cell therapy as a potential solution to tackle this problem [8,9,10,11]. 

Cell-based therapies are based on introduction of adult stem cells (peripheral blood mononuclear cells (PB-MNCs), bone marrow mesenchymal stem cells (BM-MSC), or bone marrow-derived mononuclear cells (BM-MNC) into diabetic wounds. Self-renewing and multi-differentiation are the main characteristics of these cells which underly their therapeutic advantage. Their potential to promote angiogenesis for treating diabetic microvascular and macrovascular impairments have brought stem cells to the frontier of DFU therapy. Stem cell administration is usually carried out by local injection into the wound area, which has been shown to improve healing with minimal side effects [10]. Other stem cell origins have been reported to promote wound healing in diabetes both in animal and human studies; these include adipose-derived stem cells (ADSC), endothelial progenitor cells (EPC), and mesenchymal stem cells (MSC) [12]. 

Autologous stem cells are the obvious source for cell therapies; however, studies on diabetic patients and animal models have shown that autologous SCs did not exhibit sufficient improvement of wound healing due to improper phenotype and function. Allogenic cell sources, although posing immune-compatibility issues, have been shown to successfully improve DFU healing both in animal models and clinical trials, promoting allogenic cell sources as a new therapeutic strategy [13].

Human oral mucosa exhibits a highly regenerative capacity, with fast wound-healing rates that are not affected by gender or age [4,5,14,15,16]. The basis for this unique healing and regeneration is dependent on both cellular and non-cellular factors. The oral mucosal characteristic environment defined by saliva secretion creates a humid environment which improves the survival and functioning of inflammatory cells. In addition, saliva has been shown to contain several growth factors (EFG, NGF, VEGF, TGF-β, FGF, and IGF) which play a significant role in the different stages of wound healing [17,18,19]. Accelerated wound healing has been shown to be dependent on sufficient exposure to these growth factors to promote cell migration and proliferation. Oral mucosal stem cells have been shown to produce and secrete GFs such as FGF-2, EGF, VEGF, and NGF [4] supporting the wound-healing process. hOMSCs are readily accessable and display a stable phenotype [4] over expansion, which is a clear advantage for medical and clinical use. Taken together, these unique characteristics, led us to test these cells’ ability to promote effective wound healing in diabetic wounds. We chose to test wound healing in the diabetic mouse model (*db/db* mice).

Here we show that mouse oral mucosa stem cells (mOMSCs) are highly effective in the treatment of diabetic wounds in a diabetic mouse model. Our results indicated that administration of healthy mOMSCs into diabetic wounds enhanced the rate of wound healing and re-epithelialization and the formation of granulation tissue to untreated controls.

## 2. Materials and Methods

### 2.1. Cell Culture

Isolation and culture of mOMSCs. mOMSCs were collected by harvesting the oral mucosa tissue from healthy Balb/c mice. mOMSCs were generated by explantation as previously described by us [4,20]. Briefly, the tissue obtained from the mice oral mucosa was taken out and cut into small fragments. Following 15 min incubation with dispase (2.4 U/mL) at 37 °C, the oral mucosa biopsies were washed with Dulbecco’s modified Eagle’s medium supplemented with 0.1 mg/mL streptomycin, 100 U/mL penicillin, 12.5 U/mL nystatin (SPN) (Biological Industries, Beit-Haemek, Israel), 2 mM glutamine (Invitrogen, Carlsbad, CA, USA) and 10% fetal bovine serum (Gibco, Grand Island, NY, USA) (this medium will be referred below as expansion medium). Oral epithelium was removed and the pieces of lamina propria were plated in 35 mm culture dishes (Nunc, Rochester, NY, USA) and incubated for 24 h at 37 °C and 5% CO_2_ in expansion medium. Then, explants were washed with PBS to remove debris and nonadherent cells, and fresh expansion medium was added to the cells. Expansion medium was changed every 3–4 days. Cells that emigrated from the explant to the culture dishes were harvested with 0.25% trypsin (Biological Industries, Beit-Haemek, Israel) and seeded at a cell density of 4 × 10⁴ cells per 1 cm^2^. Cells were passaged at 70–80% confluence. In order to produce sufficiently large cell stock (>10^6 cells) for transplantation, primary cells derived from oral mucosa explants needed to be passaged between 4–10 passages. Previous work in our laboratory indicated that hOMSCs’ phenotype remained stable between passages 4–11 [4]. mOMSCs were characterized similarly to hOMSC and tested for SC markers (Oct4, Sox2 and Nanog) and phenotype (supporting information in Figure S1).

### 2.2. Mouse Excisional Wound-Healing Model

Animals. All experiments were approved by the animal care committee at the Sackler Faculty of Medicine, Tel Aviv University, Israel and the Israeli Ministry of Health (M-12-071). WT mice and diabetic *db/db* mice (BKS.Cg-Dock7m +/+ Leprdb/J, #000642) were obtained from Jackson Laboratories (Bar Harbor, ME). Homozygous *db/db* mice possess a genetic mutation in the leptin receptor and represent a model of type 2 diabetes mellitus characterized by hyperglycemia, obesity, hyperinsulinemia, and impaired wound healing. These mice become obese at approximately three to four weeks of age. Elevations of plasma insulin begin at 10 to 14 days and elevations of blood sugar at 4 to 8 weeks. All of the animals used in this study were 10–12 weeks old. Mice were housed one per cage in a 12-h light/dark cycle and provided standard food and water.

PCR genotyping of mice tail DNA. Tail DNA was obtained from 14 day-old, heterozygous F2 littermates. Genotyping for WT and diabetic mice was carried out by PCR (KAPA_Mouse_Genotyping_Kit_TDS, KAPA biosystems), using primers specific for the wild-type and the mutant alleles and a common primer (WT- AGC CAC TAC AAT CCA CCC CTT G; Mutant- GCA GTG CAC AGG CTC AGG AA; Common- GCT GCA GAA TGG ACG GTT GA). The PCR data were verified by the color of the mice, the blood sugar levels, and the weight.

Diagnosis of diabetes. Diabetic mice (*db/db* genotype) were defined as those with confirmed hyperglycemia, expressing non-fasting blood glucose levels > 250 mg/dL from tail vein (Contour, Bayer).

Preparation of stem cells for transplantation. Cells for transplantation were collected from cultures at 80–90% confluency. Cells were washed with PBS, harvested with trypsin-EDTA, counted and resuspended at a concentration of 3 × 10^6^ in 200 µL in PBS. To prevent aggregation, cells were prepared immediately before transplantation.

Experimental wound-healing model. A model of excisional skin wound healing described previously by Galiano et al. [21], was used with changes detailed hereinafter. Briefly, WT and *db/db* mice were anesthetized using inhalation of isoflurane. Hair was shaved from the dorsal side of each animal. The skin surface to be excised and its surrounding area were rinsed with povidone-iodine 10%. A pain-relief subcutaneous injection (Rimadyl) was administered. In order to prevent skin contraction at the wound site, a doughnut-shaped silicone splint with an 8 mm inner diameter was sutured to the skin by 4-0 nylon continuous sutures. A sterile 6 mm diameter biopsy punch tool (Kruse) was used in the center of the silicone splint to make a full-thickness wound extending through the panniculus carnosus. The animals were housed in the institutional animal facility. Animals were divided into 4 groups: WT PBS-treated (control), WT PBS + mOMSCs-treated, *db/db* PBS-treated (control), and *db/db* PBS + mOMSCs-treated, each consisting of 6 to10 mice per group.

In the treated groups 200 µL PBS containing 3 × 10^6^ suspended mOMSCs generated from the lamina propria of the oral mucosa of allogeneic Balb/c mice was injected (insulin syringe, 30G) subdermally around and under the wound. In the control groups 200 µL PBS injections were injected at the same sites as in the treated group. Wounds were dressed with Tegaderm sterile dressing (3M Healthcare, St Paul, MN, USA).

Luciferase-labeled mOMSCs were injected into the wound. Cells were monitored on days 1, 7, and 14 days post-wounding using in vivo bioluminescence imaging performed after the luciferin administration.

Following surgery, the mice were placed in individual cages under a lamp, and allowed to fully recover from anesthesia.

Wound morphology analysis. Digital photographs of the wounds (Nikon camera) were taken at the time of surgery and every 3–4 days thereafter until wound closure. Wounds were considered as closed when the wound bed was completely filled in with new tissue. The wound area was measured at each time point using ImageJ software (NIH, Bethesda, MD, USA). The global wound area (%) was calculated according to the residual wound area on a given day (tx) relative to the wound area measured on the day of surgery. A wound was considered to be completely closed when the wound area was equal to zero. The rate of wound closure in the treated *db/db* was monitored and compared to control animals.

Histomorphometry. We analyzed histological sections of PBS + mOMSCs-treated and PBS-treated diabetic animals (n = 5 per time point) at 0, 7, and 11 days following wounding.

Histomorphometry of the Epithelial Gap. At the time of sacrifice, wounds were excised, and fixed in 10% formalin for 6 days. The samples underwent routine histological processing for paraffin embedding. The tissue was sectioned and stained with hematoxylin and eosin (H&E). All histological sections were scanned using Aperio ImageScope. The epithelial gap (EG) was digitally analyzed and defined as the distance between the advancing edges of epidermal keratinocytes. Three to six serial sections were averaged to determine the EG at each time point. An EG of zero represented a completely re-epithelialized wound.

Granulation tissue staining. The histological images were analyzed for the total area of GT using digital analysis software. The area of GT was calculated by tracing regions of GT and calculating pixel area. The total area of granulation was the sum of these regions.

Statistical Analysis. Results are expressed as mean ± SEM. Unpaired, two tails Student’s *t*-test and one-way analysis of variance for multiple comparisons with Tukey post hoc test were used to determine statistical significance, which was defined by a *P* value < 0.05.

## 3. Results

### 3.1. mOMSCs Augment Wound Healing in Diabetic Mice—Global Wound Area

**Figure 1:**
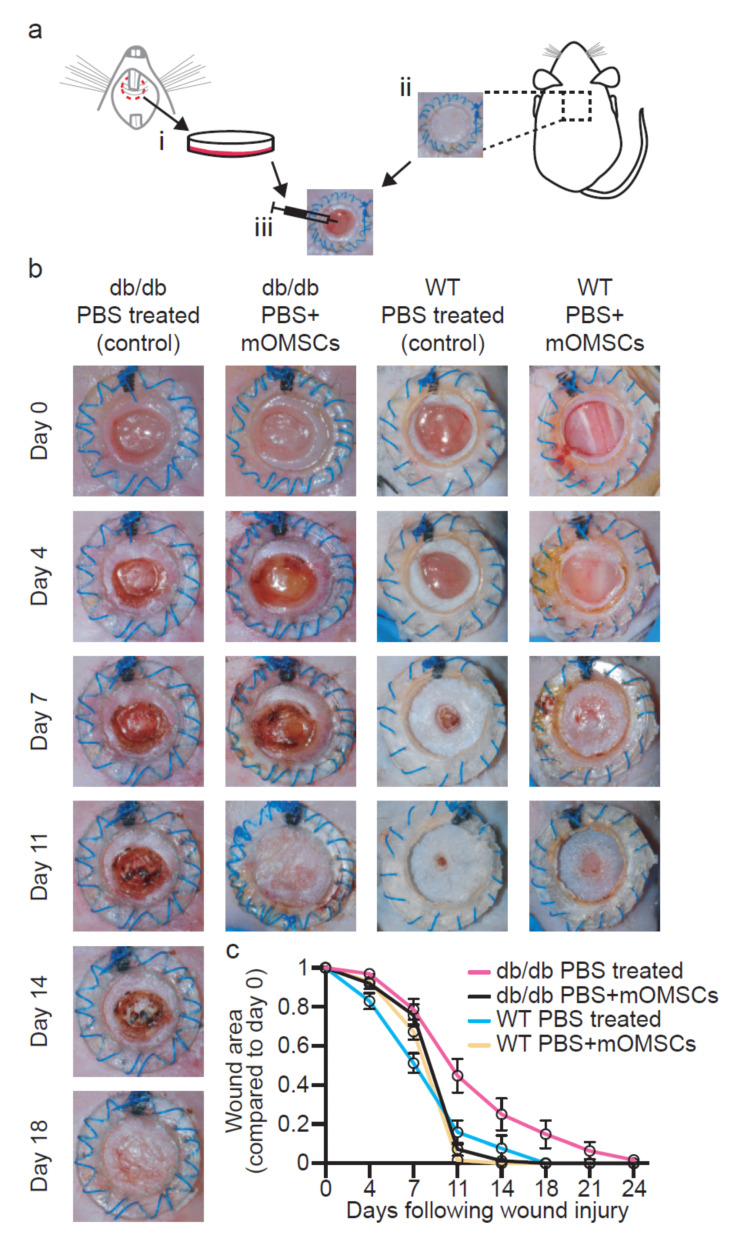
(**a**) Schematic drawing of wound-healing model for the evaluation of the therapeutic potential of mouse oral mucosa stem cells (mOMSCs). i. mOMSCs were isolated from oral mucosa tissue of healthy Balb/c mice. ii. Wound excision. iii. Oral mucosa stem cells of allogeneic Balb/c mice were injected around and under the wound. (**b**) Change in wound area. Digital photographs showing the change in the wound area over time in WT and *db/db* PBS + mOMSCs-treated and PBS-treated mice. (**c**) Kinetics of wound closure. Differences in mean wound area of treated WT and *db/db* mice with Balb/c mOMSCs + PBS (yellow and black, respectively) and PBS-treated WT and *db/db* mice (blue and pink, respectively). Each point represents the mean of the percentage in area of the original wound size ± SEM (*p* < 0.05).

Wounds were inflicted onto the skin of type II diabetes mice (i.e., *db/db*) and WT mice. Wounds were treated with stem cells isolated from the lamina propria of the oral mucosa of healthy Balb/c mice (Figure 1a). Wound healing was assessed macroscopically at the following time points: 0, 4, 7, 11, 14, 18, 21, and 24 days post-wounding or until complete wound closure. The results demonstrate that mOMSCs administration did not accelerate wound healing in WT animals (Figure 1b). However, in diabetic wounds, mOMSCs administration resulted in a significantly increased rate of wound closure as compared to control diabetic wounds treated with PBS, particularly at day 11 (*p* ≤ 0.0001) and 14 (*p* ≤ 0.0123) following wounding (Figure 1b,c). Notably, the rate of wound closure in the mOMSCs-treated diabetic mice followed a similar wound-healing course as that of WT controls. Moreover, while most of the wounds in diabetic mice treated with mOMSCs were closed by day 11 (67%), and all the wounds were fully closed by day 14, PBS-treated diabetic wounds exhibited wound closure by day 18 (55%), and some of the wounds were closed only 24 days after wounding.

**Figure 2:**
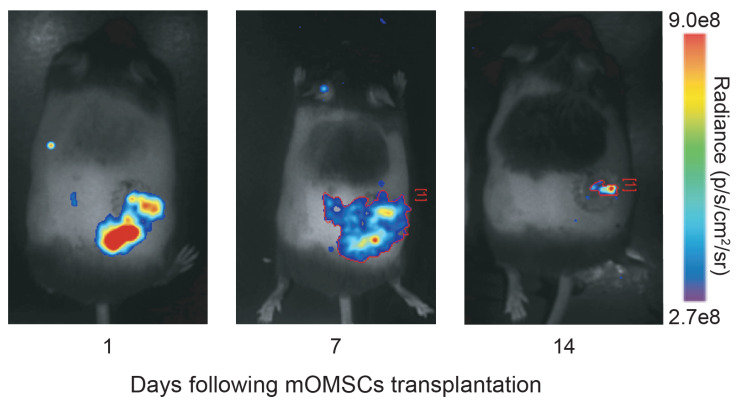
Luciferase-labeled mOMSCs remain viable in the wound area. In vivo bioluminescence imaging of mice 1, 7, and 14 days following administration of Luciferase-labeled mOMSCs to the wound. In this assay, only viable cells express luciferase, which emits light following chemical conversion of luciferin, allowing monitoring of the viability, location, and concentration of the luciferase-labeled cells.

To test whether the allogeneic mOMSCs survived the transplantation, luciferase-labeled mOMSCs were administered to the wound area. In this assay only viable cells that express luciferase are able to react with luciferin and become fluorescent. The data shown in Figure 2 indicates that some of the luciferase-labeled allogeneic mOMSC remained viable in the wound area for at least 14 days.

### 3.2. Wound-Healing Histology

**Figure 3:**
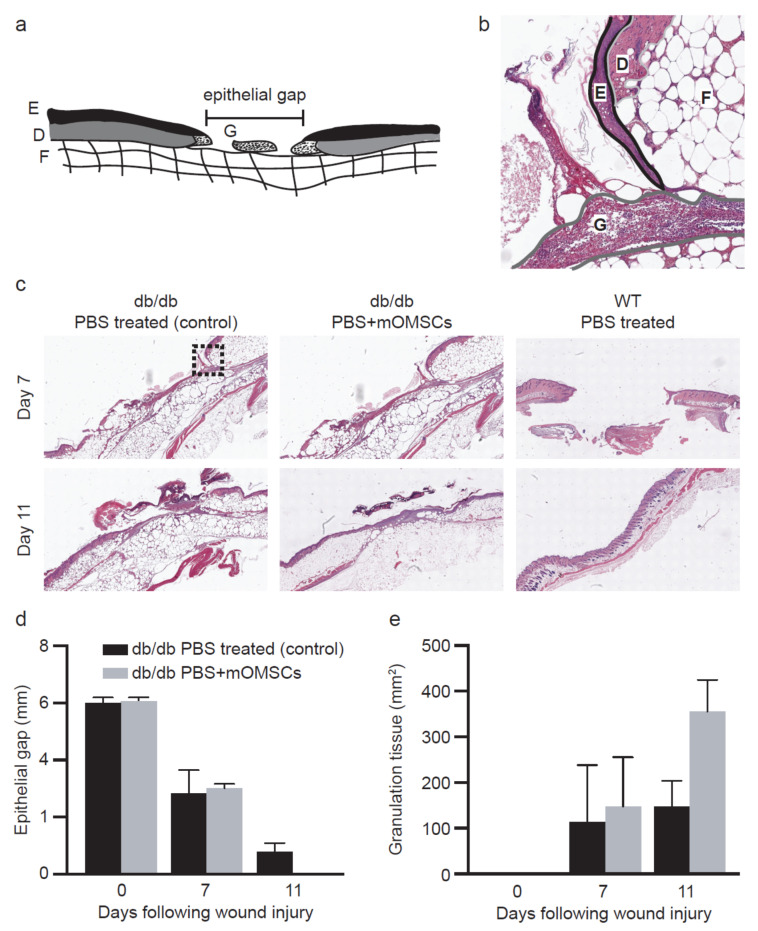
(**a**) Schematic drawing of histological section of the wound model for evaluation of wound healing. Epithelial gap (EG) and total area of granulation tissue were analyzed using digital image analysis software. E = epithelium, G = granulation tissue, D = dermis, F = fat. Epithelial gap was analyzed using digital image analysis software. Granulation tissue area was measured by tracing regions of granulation tissue and calculating pixel area. The sum of the regions was defined as the GT area. (**b**) Histological section of wound in db/db mice. E = epithelium, G = granulation tissue, D = dermis, F = fat. (**c**) Histological comparison of epithelial gap and granulation tissue formation in PBS-treated and PBS + mOMSCs-treated db/db mice and WT control. (**d**) Comparison of epithelial gap in PBS + mOMSCs-treated and PBS-treated db/db mice. EG was assessed at days 0, 7 and 11 after wounding, H&E staining. (**e**) Effect of treatment on the rate of GT formation in db/db mice as assessed at days 0, 7 and 11 after wounding

To further investigate the cellular basis underlying the wound morphology findings, we examined histological sections of the wounds of mOMSCs-treated and diabetic controls (PBS only). Specifically, we measured the size of the epithelial gap and the area of granulation tissue, indicative of epithelial closure and healing capacity of the tissue, respectively (Figure 3a,b). Wound healing was examined at specific time points to map treatment effectiveness. We found that 11 days following wounding, the epithelial gap in mOMSCs-treated *db/db* animals was significantly smaller compared to PBS-treated diabetic controls (*p* = 0.0354) (Figure 3c,d). Furthermore, granulation tissue area was more abundant at this time point (day 11) in mOMSCs-treated diabetic animals (*p* = 0.0347) (Figure 3e) indicating a more advanced healing stage. Earlier tested time points did not show statistical significance of healing rate between mOMSCs-treated and diabetic controls (data not shown).

## 4. Discussion

The wound-healing process involves cascades of molecular and cellular events. This process includes overlapping phases of hemostasis, inflammation, proliferation and tissue remodeling [22,23,24]. Growth factors including FGF, VEGF, and EGF play a critical role in initiating and sustaining the different phases of wound healing. These factors are responsible for re-epithelialization, angiogenesis, granulation tissue formation, wound contraction, and synthesis of new extracellular matrix. Diabetic chronic wounds show delayed wound healing which has been linked to changes in amount and type of growth factors due to reduction in their expression, production and secretion, trapping, and rapid degradation [8].

A thorough understanding of the causes for impaired wound healing in diabetes will lead to the development of several treatment approaches. To date, the standard care therapies are offloading and mechanical or biological wound debridement [25]. However, these treatments do not target the problematic etiology and are aimed at treatment of the wound symptoms and/or complications. Recently, in vitro and preclinical studies for adjunctive treatments for diabetic wound care have included recombinant growth factor, cell- and tissue-based products (CTPs), acellular matrices, platelet-rich plasma, and other biologics therapies [8,25]. However, few have been proven to induce complete ulcer healing [26]. One of the most promising therapeutic concepts for wound healing is based on stem cells which have shown the potential to regenerate tissue to its pre-injured state [11,26]. However, the field of therapy of the most common stem cells, ESC and iPSC, involves ethical considerations and genetic manipulation. Mesenchymal stromal cells (MSC) have gained popularity as an alternative stem cell source for cell-based therapies [27] and have been shown to accelerate wound healing by promoting angiogenesis and modulating the immune response [28].

We found that injection of mOMSCs into the wounds of diabetic *db/db* mice induced faster wound healing. While previous findings demonstrated that in similar models, injection of allogeneic ADSCs with hydrogel led to enhanced wound closure by day 14.6 ± 1.1 post-wounding [29], most (67%) of our mOMSCs treated wounds were closed by day 11 (Figure 1b,c). Moreover, we showed that mOMSCs-treated diabetic wounds exhibited healing rates similar to non-diabetic WT mice. We showed that the wound-healing process promoted by mOMSCs followed normal regulated healing since WT wounds injected with mOMSCs did not exhibit changes in wound-healing rate (Figure 1b,c). Studies have shown that allogeneic transplantation provides a healthy source of SCs, established in both preclinical and clinical trials, with no risk of cell harvesting for the diabetic patient [30]. Further investigation of the wound-healing potential of OMSCs derived from diabetic origin (autologous explants) should be the focus of future preclinical and clinical studies.

Humans and rodents have different wound-healing mechanisms. While wounds in humans heal mainly by re-epithelialization and granulation tissue formation, rodents display contraction-based wound healing [21]. Therefore, a silicone splint was bonded and sutured to the skin surface, allowing gradual wound closure to occur by re-epithelialization and granulation tissue formation. We showed that the epithelial gap of the mOMSCs-treated animals was closed by day 11 while none of the untreated control wounds exhibited wound closure. This finding was further supported with histological analysis of the wounds which showed larger areas of granulation tissue. These results point to the contribution of mOMSCs to the wound repair processes as, for example, re-epithelialization and granulation formation.

Regenerative strategies based on MSCs describe the paracrine signaling, through GF secretion, as the main mechanism by which MSCs induce accelerated wound healing [31]. Based on studies showing that hOMSCs secrete FGF-2, EGF, VEGF, and NGF in vitro [4], it is suggested that in our study, the subdermal injection of mOMSCs into the wounds and at their periphery induced accelerated wound closure through paracrine secretion of GFs. Further investigation into the molecular mechanism of mOMSCs signaling and the reciprocal cellular responses in vivo are needed.

The highly beneficial characteristics of OMSCs, such as accessibility, ease of isolation, high numbers and availability throughout life with minimal host age effects, suggest that these cells can be regarded as a promising therapeutic cellular tool for tissue repair. We believe that the use of OMSCs in delayed wound healing such as in chronic diabetic wounds can be considered a promising and valid clinical application. We believe that autologous OMSCs will show similar wound-healing properties when tested in chronic and diabetic wounds. Further preclinical studies are needed to assess the safety and efficacy of human-derived OMSC.

Even though still at the preclinical level, stem cell therapy is a promising therapeutic tool for the treatment of diabetic wounds in humans. Nevertheless, the translation from animals to humans is expected to encounter considerable hurdles. The main reason for this is the major difference between the morphology and physiology of animal and human skin. Therefore, the therapeutic value of stem cells in general and that of OMSC in particular for diabetic wound healing treatment awaits to be assessed in human clinical studies.

## 5. Conclusions

We found that the injection of mOMSCs into full-thickness chronic diabetic wounds accelerated wound healing to a rate similar to that of healthy WT animals. Our results point to mOMSCs as key potential allogeneic therapeutic agents for treating diabetic wounds.

## Supplementary Materials

The following are available online at https://www.mdpi.com/1660-4601/17/13/4854/s1, Figure S1: Immunophenotype of Balb/c mouse oral mucosa stem cells (mOMSCs).
