# Supplementary material for: Examination of the Therapeutic Potential of Mouse Oral Mucosa Stem Cells in a Wound-Healing Diabetic Mice Model
Source: Int J Environ Res Public Health. 2020 Jul 6;17(13):4854. doi: 10.3390/ijerph17134854 (PMC7369976; doi:10.3390/ijerph17134854)
Supplement: Supplementary file 1 [file ijerph-17-04854-s001.pdf]

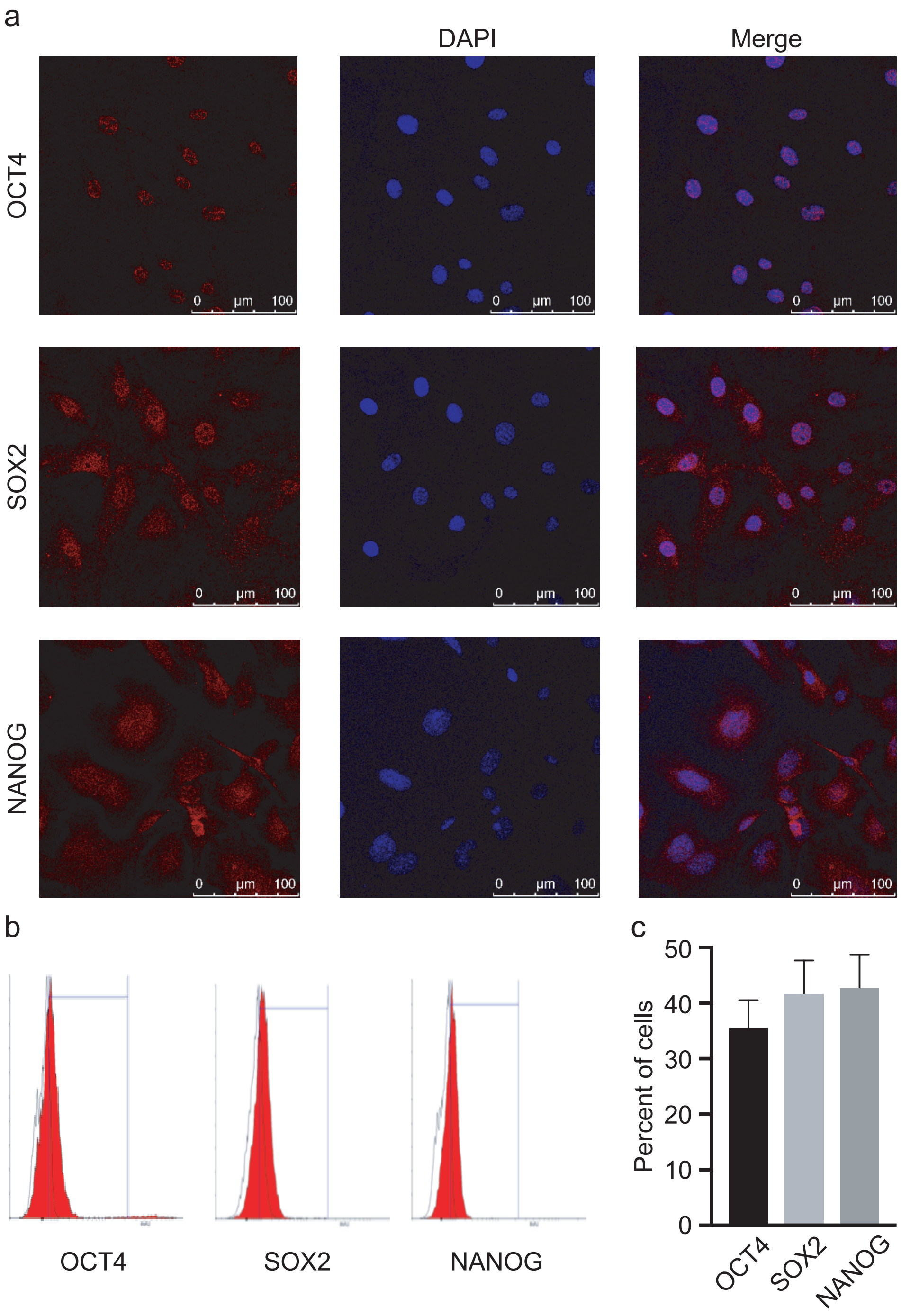

Fig. 1S.

Immunophenotype of Balb/c mouse oral mucosa stem cells (mOMSCs). **(a)** Immunofluorescent staining of intracellular stem cell markers. Similarly to human oral mucosa stem cells (hOMSCs), mOMSCs express the pluripotency markers Oct4, Sox2 and Nanog. **(b)** Flow cytometry analysis of the pluripotency-markers- Oct4, Sox2 and Nanog observed in Balb/c-derived mOMSCs. **(c)** The percentages of cells positive for these markers are similar to that observed in hOMSC.
